# Patterns of pesticide usage in agriculture in rural Tanzania call for integrating agricultural and public health practices in managing insecticide-resistance in malaria vectors

**DOI:** 10.1186/s12936-020-03331-4

**Published:** 2020-07-16

**Authors:** Nancy S. Matowo, Marcel Tanner, Givemore Munhenga, Salum A. Mapua, Marceline Finda, Jürg Utzinger, Vera Ngowi, Fredros O. Okumu

**Affiliations:** 1 Environmental Health & Ecological Sciences, Ifakara Health Institute, Ifakara, Tanzania; 2 Swiss Tropical and Public Health Institute, Basel, Switzerland; 3 University of Basel, Basel, Switzerland; 4 Department of Disease Control, London School of Hygiene and Tropical Medicine, London, UK; 5 Wits Research Institute for Malaria, School of Pathology, University of the Witwatersrand, Johannesburg, South Africa; 6 Centre for Emerging Zoonotic and Parasitic Diseases, National Institute for Communicable Diseases, Johannesburg, South Africa; 7 School of Public Health, University of the Witwatersrand, Johannesburg, South Africa; 8 Muhimbili University of Health and Allied Sciences, Dar es Salaam, Tanzania; 9 Institute of Biodiversity, Animal Health and Comparative Medicine, University of Glasgow, Glasgow, UK; 10 School of Life Science and Bioengineering, Nelson Mandela African Institution of Science and Technology, Arusha, Tanzania

**Keywords:** Malaria Vector, Agricultural practices, Lambda-cyhalothrin, Chlorpyrifos, Chlorothalonil, Imidacloprid, Glyphosate, Pesticides knowledge, Insecticide resistance, Malaria

## Abstract

**Background:**

Unrestricted use of pesticides in agriculture is likely to increase insecticide resistance in mosquito vectors. Unfortunately, strategies for managing insecticide resistance in agriculture and public health sectors lack integration. This study explored the types and usage of agricultural pesticides, and awareness and management practices among retailers and farmers in Ulanga and Kilombero districts in south-eastern Tanzania, where *Anopheles* mosquitoes are resistant to pyrethroids.

**Methods:**

An exploratory sequential mixed-methods approach was employed. First, a survey to characterize pesticide stocks was conducted in agricultural and veterinary (agrovet) retail stores. Interviews to assess general knowledge and practices regarding agricultural pesticides were performed with 17 retailers and 30 farmers, followed by a survey involving 427 farmers. Concurrently, field observations were done to validate the results.

**Results:**

Lambda-cyhalothrin, cypermethrin (both pyrethroids) and imidacloprids (neonicotinoids) were the most common agricultural insecticides sold to farmers. The herbicide glyphosate (amino-phosphonates) (59.0%), and the fungicides dithiocarbamate and acylalanine (54.5%), and organochlorine (27.3%) were also readily available in the agrovet shops and widely used by farmers. Although both retailers and farmers had at least primary-level education and recognized pesticides by their trade names, they lacked knowledge on pest control or proper usage of these pesticides. Most of the farmers (54.4%, n = 316) relied on instructions from pesticides dealers. Overall, 93.7% (400) farmers practised pesticides mixing in their farms, often in close proximity to water sources. One-third of the farmers disposed of their pesticide leftovers (30.0%, n = 128) and most farmers discarded empty pesticide containers into rivers or nearby bushes (55.7%, n = 238).

**Conclusion:**

Similarities of active ingredients used in agriculture and malaria vector control, poor pesticide management practices and low-levels of awareness among farmers and pesticides retailers might enhance the selection of insecticide resistance in malaria vectors. This study emphasizes the need for improving awareness among retailers and farmers on proper usage and management of pesticides. The study also highlights the need for an integrated approach, including coordinated education on pesticide use, to improve the overall management of insecticide resistance in both agricultural and public health sectors.

## Background

The control of malaria and other vector-borne diseases relies primarily on insecticide-based interventions, such as long-lasting insecticidal nets (LLINs) and indoor residual spraying (IRS) [1, 2]. The effectiveness of these interventions is being compromised by the increased geographical spread of insecticide in the targeted mosquito populations [3, 4]. Insecticide-resistance by mosquito populations to the limited number of insecticides approved for vector control has been implicated as the key driver of persistent malaria transmission [5, 6].

Insecticide resistance in malaria vectors is predominantly attributed to exposure of mosquitoes to public health insecticides [3, 4]. However, agricultural pesticides also exert strong selection pressures, thus contributing to resistance in vector species [7–14]. This is because of similarities in chemicals used, applications of these chemicals simultaneously, and their indiscriminate use in agriculture [15]. This phenomenon was observed in West Africa where *Anopheles gambiae* sensu lato (s.l.) populations sampled from farmlands characterized by high agriculture pesticide usage showed higher levels of resistance to insecticides compared to populations sampled in areas with limited or no agricultural pesticide usage [11–13, 16]. Similarly, in Sudan agricultural usage of organophosphate and carbamates was linked to insecticide resistance in *Anopheles arabiensis* [17]. Aquatic exposures of mosquito larvae to sub-lethal doses of pesticides, herbicides and other pollutants have also been linked to higher tolerance to insecticides in malaria vectors [9, 18–20]. Furthermore, Chouaïbou et al. found that over 90% of the insecticides used by vegetable and rice farmers in the southern part of Côte d’Ivoire were pyrethroids similar to those approved for vector control [21].

In many malaria endemic countries, agriculture is the main economic activity. To improve crop yields in these regions there is the rampant use of pesticides, fungicides and herbicides [22–24]. For example, in Tanzania, approximately 81% of pesticides are deployed in both agricultural and veterinary sectors [25]. Concurrently, pyrethroid impregnated LLINs are also widely used against disease vectors in these regions.

The World Health Organization (WHO) Global Malaria Programme has developed a global action plan for insecticide resistance management in malaria vectors to preserve the effectiveness of LLINs and IRS [26]. The principal recommended resistance management approaches, mostly adopted from agriculture include: (i) annual rotation of insecticides with different modes of action; (ii) combination of pyrethroid-based LLINs and IRS with non-pyrethroids; (iii) mosaic spraying of two different insecticide classes in different geographical locations; and (iv) mixtures of different classes of insecticides into a single product [26]. However, resistance management policies have yet to be integrated into agricultural and disease control programmes. As a result, the programmes do not account for the collective contributions by both public health and agricultural sectors to the spread of insecticide resistance.

The purpose of this study was to explore agricultural pesticides, pesticide usage practices, awareness, and management practices among retailers and farming communities from a rural malaria endemic area in south-eastern Tanzania, where mosquito vectors are resistant to public health insecticides [27, 28]. The findings are expected to guide practical recommendations for collaboration between agriculture and public health sectors in insecticide resistance management in mosquito vectors and disease control.

## Methods

### Study area

**Fig. 1:**
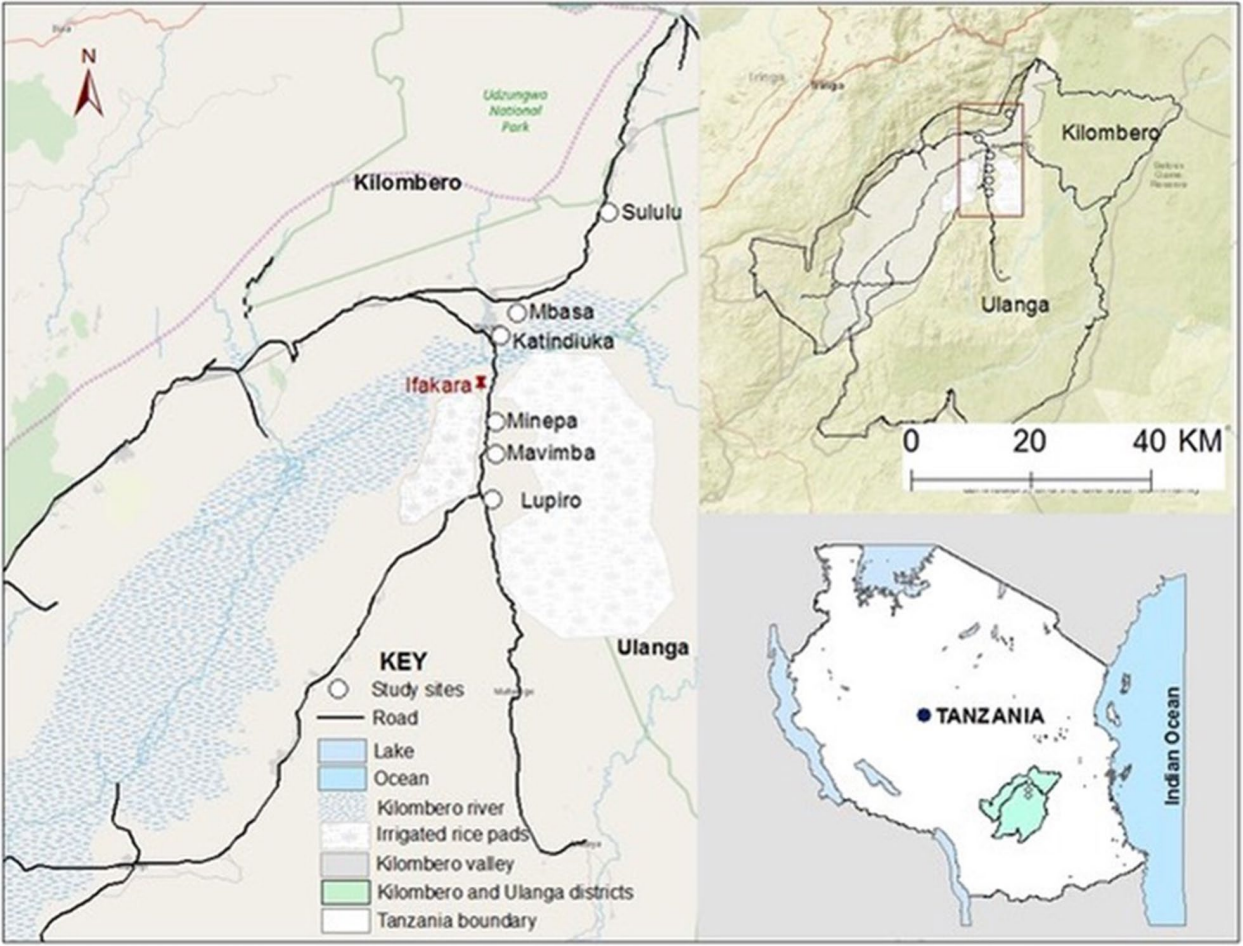
Map of south-eastern Tanzania showing the study wards in the districts of Kilombero and Ulanga in the Kilombero Valley

The study was conducted in six wards, in Kilombero and Ulanga districts, south-eastern Tanzania (altitude ~ 300 m; annual precipitation: 1200–1800 mm; temperatures: 20–32 °C), purposefully selected to represent different agro-ecological areas (Fig. 1). Rice farming is the main economic activity of the area [29]. Vegetable and fruit cultivation is also quite common. Farmers here widely use synthetic pesticides and chemical fertilisers. During the dry season, rice production is maintained by irrigation (locally known as “*Ngapa*”) rendering the area continuously favourable for mosquitoes [30]. Malaria burden remains significant, with the heaviest burden experienced in children below 5 years [31, 32]. *Anopheles funestus* sensu stricto (s.s.) and *An. arabiensis* are the predominant malaria vectors [27, 28]. Additionally, non-malaria vectors, such as *Culex* and *Mansonia,* constitute biting nuisances [33, 34]. Though pyrethroid-based LLINs are the main malaria intervention [35], mosquito populations are resistant to pyrethroids, bendiocarb (carbamates), and DDT [27, 28, 34].

### Study design and data collection

An exploratory sequential mixed-methods study design was employed. In-depth interviews were done for collecting qualitative data and structured questionnaires were used to collect quantitative data (Additional file 1). Both data collection tools were prepared in English, translated and used in Kiswahili the local native language. The questionnaires were pre-tested on a few participants (who were not otherwise enrolled in the actual study) to ensure clarity before the actual study. Direct observations were made and photographs taken of the pesticides in the stores to identify their active ingredients, and handling practices. In the farms prior observations were validated on pesticides usage and handling practices. Data collection was conducted between February 2017 and November 2017.

#### Exploration of awareness and perceptions of pesticides use, storage and disposal

In-depth interviews were conducted with agricultural and veterinary (agrovet) retail stores (n = 17) and with famers in the six wards (n = 30). With the retailers, the interviews aimed to explore awareness of pesticide prescription and handling practices. Interview guides explored the retailers’ awareness and perceptions of (i) types of agricultural pesticides, knowledge of pesticides sold at their shops; and (ii) source of knowledge on using the pesticides, pesticides preferences, frequency of purchases and seasonal use of the pesticides/frequency of applications. With the community members, the interviews explored awareness and perceptions regarding different agricultural pesticides, use and storage methods, and challenges faced. Direct observations of agricultural practices in the farms, including handling and disposal practices of the pesticides were also done. Initial findings from these qualitative studies informed subsequent quantitative studies. All interviews were audio-recorded and field notes taken by the data collector.

#### Assessment of knowledge and practices regarding pesticide use

A cross-sectional survey using an electronic questionnaire form in an Open Data Kit (ODK) [36] was conducted with 427 randomly selected farmers from the six wards. The questionnaire assessed the farmers’ awareness and practices of agricultural pesticides use, storage and disposal. Findings from the qualitative and quantitative study and direct observations were triangulated.

#### Assessment of types and classes of agricultural pesticides

Direct observations of the agricultural pesticides were done at all of the 17 agrovet retail stores. Information collected included pesticide types, classes and active ingredients.

### Analysis of qualitative and quantitative data

Audio recorded interviews with the retailers of agricultural pesticides and farmers were transcribed verbatim and translated to English. The transcripts were imported into MAXQDA software for coding and analysis [37]. Systematic review and analysis of key issues, concepts, and repeated themes were done following framework analysis steps as described by Gale and colleagues [38]. For the data from farmers, a weaving approach was used, in which both quantitative and qualitative components were presented together [39]. Quantitative findings from the survey were presented, and further explanations drawn from the in-depth interviews. Selected participant’s narratives from each theme are presented.

Quantitative data generated through surveys from agrovet stores were analysed descriptively, using Stata version 15 (Stata Cooperation; College Station, TX, USA). Pictures of all of the insecticides were individually reviewed and active chemical ingredients recorded to summarize their frequencies by insecticide class.

## Results

### Characteristics of pesticide retailers and farmers

More than half (58.8%, n = 10) of the agrovet stores were in Kilombero district, while the remaining 41.2% (n = 7) were in Ulanga district. Two-thirds of participants (65.2%, n = 11) were females with age ranging between 18 and 43 years.

Table 1 summarizes the demographic characteristics of the farmers who participated in the survey. Males comprised of 51.5% (n = 220) and females 48.4% (n = 207). Most farmers practised both small-scale subsistence farming 51.3%, (n = 219) and large-scale cultivation 48.5% (n = 207) for food and business, and had worked on their farms for at least 5 years 89.2% (n = 381). The main farm crops farmed were rice, maize, different types of vegetables and fruits.

**Table 1:** Socio-demographic characteristics of farmers involved in the survey

Variable	Category	Percentage (n)
Gender	Males	51.5% (220)
	Females	48.5% (207)
Age (years)	18–30	16.9% (72)
	31–40	31.1% (133)
	41–50	28.3% (121)
	51–60	17.6% (75)
	> 60	6.1% (26)
Education attainment	Primary school	85.2% (364)
	Secondary school	9.6% (41)
	College/university	0.7% (3)
	Professional training	0.5% (2)
	No formal training	4.0% (17)
Main economic activities^a^	Small-scale subsistence farming activities	51.3% (219)
	Large-scale farming for food and business	48.5% (207)
	Livestock keeping	9.8% (12)
	Small-scale business	41.7% (178)
	Large-scale business	2.8% (3)
	Private employment	0.7% (2)
	Others	0.5% (42)

^a^Farmers with more than one sources of income, multiple responses

### Types and classes of agricultural pesticides

The agricultural pesticides (Additional file 1), chemical classes and the active ingredients observed in the agrovet stores are summarized in Table 2. Most of the agricultural pesticides (87.5%, n = 91) were approved plant protection substances under full registration category (6.7%, n = 7) or had restricted registration or provisional registration according to Tanzania regulations [40, 41]. A small proportion (2.9%, n = 3) were unregistered. Insecticides accounted for (59.6%, n = 62) of the pesticides, followed by herbicides (27.9%, n = 29) and fungicides (10.6%, n = 11). The highest proportion of agricultural insecticides surveyed were organophosphates (34%), followed by pyrethroids (30%). Herbicides from the amino-phosphonates class were the most popular (59%). The two main fungicide classes were dithiocarbamate (54.5%) and acylalanine organochlorine (27.3%), widely used by most vegetable growers (Table 2). The insecticide formulations were emulsifiable concentrate (EC) (63%), while (66%) herbicides, and (64%) fungicides were formulated as soluble (liquid) concentrate (SL) and wettable powders (WP), respectively (Additional file 2).

**Table 2:** Common active ingredients found in the agricultural pesticides in the study locality

Pesticide type	Active ingredient (s)	N	%	Chemical class
Insecticides (N = 62)	Abamectin	4	6.5	Macrocyclic lactones
	Alphacypermethrin	3	4.8	Pyrethroids
	Carbaryl and permethrin	1	1.6	Carbamates and pyrethroids
	Carbofuran	1	1.6	N-methyl carbamate Ib
	Carbaryl and lambda-cyhalothrin	2	3.2	Carbamates and pyrethroids
	Chlorpyrifos	5	8.1	Organophosphates
	Cypermethrin	1	1.6	Pyrethroids
	Cypermethrin and chlorpyrifos	1	1.6	Pyrethroids and organophosphates
	Cypermethrin and imidacloprid	3	4.8	Pyrethroids and neonicotinoids
	Deltamethrin	1	1.6	Pyrethroids
	Diazinon	2	3.2	Organophosphates
	Dichlorvos	3	4.8	Organophosphates
	Dimethoate	1	1.6	Organophosphates
	Fenitrothion and deltamethrin	3	4.8	Organophosphates and pyrethroids
	Fipronil	1	1.6	Phenylpyrazole
	Imidacloprid	3	4.8	Neonicotinoids
	Imidacloprid and beta-cyfluthrin	2	3.2	Neonicotinoids and pyrethroids
	Lambda-cyhalothrin	11	17.7	Pyrethroids
	Lambda-cyhalothrin and acetamiprid	1	1.6	Neonicotinoids and pyrethroids
	Malathion	1	1.6	Organophosphates
	Permethrin	1	1.6	Pyrethroids
	Pirimiphos-methyl	2	3.2	Organophosphates
	Pirimiphos-methyl and permethrin	3	4.8	Organophosphates and pyrethroids
	Pirimiphos-methyl and thiamethoxam	1	1.6	Organophosphates and neonicotinoids
	Profenofos	5	8.1	Organophosphates
Herbicide (N = 29)	Bispyribac sodium	1	3.5	Bispyribac sodium
	S-metolachlor and atrazine	1	3.5	Triazines
	Amine salt	4	13.8	Aryloxyacides
	Atrazine	1	3.5	Dinitroanilines
	Glyphosate	17	58.6	Amino-phosphonates
	Paraquat	4	13.8	Pyridines
	Triclopyr	1	3.5	Pyridines
Fungicide (N = 11)	Monopotassium and dipotassium phosphonates	1	9.1	Phosphonic acid
	Chlorothalonil	3	27.3	Organochlorine
	Mancozeb	1	9.1	Dithiocarbamate
	Mancozeb and cymoxanil	1	9.1	Acylalanine and dithiocarbamate
	Metalaxyl and mancozeb	5	45.5	Dithiocarbamate and acylalanine
Insecticide + fungicide (N = 2)	Imidacloprid, metalaxyl and carbendazim	2	100	Neonicotinoids, acylalanine and benzimidazole

Most insecticides had a single active ingredient (72.6%, n = 45), while fewer were mixed products with two different active ingredients at different doses (27.4%, n = 17), as shown in Tables 2 and 3. The most common pyrethroid was lambda-cyhalothrin, while chlorpyrifos and profenofos were the predominant organophosphates (Table 2). Most of the insecticides are non-systemic broad-spectrum insecticides with contact and stomach actions against crop pests. Over half of the herbicides (59%) were based on glyphosate that were frequently used by most of the rice farmers (76.8%). The principle active ingredients in most fungicide were metalaxyl and mancozeb (45%) and chlorothalonil (27%) (Table 2). Table 3 summarizes some of the commonly used pesticide products with more than one active ingredients. A wide range of insecticide classes and active ingredients used in crop protection had similar target sites and modes of action with the limited public health insecticides (Table 4).

**Table 3:** Example of pesticide products with more than one active ingredient (as obtained from the factory)

WHO class/family	Brand name	Active ingredient(s)
Organophosphates and pyrethroids	Simba powder 113DP	10 g/kg of fenitrothion and 1.3 g/kg of deltamethrin
	Duduba 450EC	350 g/l of chlorpyrifos and 100 g/l of cypermethrin
	Mupa dust	1.0% of fenitrothion and 0.13% of deltamethrin
	Stocal super dust	16 g/kg of pirimiphos-methyl and 3 g/kg of permethrin
	Shumba super dust	1% of fenitrothion and 0.13% of deltamethrin
	Actellic Gold Dust	16 g/kg of pirimiphos-methyl and 3.6 g/kg of thiamethoxam
	Haigram 90 dusting powder (DP)	6 g/kg of pirimiphos-methyl and 3 g/kg of permethrin
	Actellic super dust	16 g/kg of pirimiphos-methyl and 3 g/kg of permethrin
Pyrethroids and neonicotinoids	Amekan C344 SE	144 g/l of cypermethrin and 200 g/l of imidacloprid
	Rapid-attack 344SE	144 g/l of cypermethrin and 200 g/l of imidacloprid
	Blast 60 EC	3% g/l lambda-cyhalothrin and 3% g/l of acetamiprid
	Buffalo 450OD	2.5% of beta- cyfluthrin and 7.5% of imidacloprid
	Thunder Oil Dispersion (OD) 145	45 g/l of beta-cyfluthrin and 100 g/l of imidacloprid
	Farmguard 344SE	144 g/l of cypermethrin and 200 g/l of imidacloprid
Carbamates and pyrethroids	Bakiller	5% w/w of carbaryl and 0.1% w/w of lambda cyhalothrin
	Akheri powder	5% w/w carbaryl and 0.1% w/w lambda-cyhalothrin
	Ultravin^®^ Dudu dust	5% w/w of carbaryl, 1% w/w of permethrin and 94% w/w of inert carriers
Neonicotinoids, acylalanine and benzimidazole	Seed plus 20 wettable soluble (WS)	10% imidacloprid, 5% metalaxyl and 5% carbendazim WS

**Table 4:** Similarities between agricultural and public health insecticide classes and reported resistance mechanisms in disease vectors

Class of insecticide	Trade name (active ingredient (s)	Primary site/mode of action in an insect/vector	Agricultural use	Public health use	Known resistance and resistance mechanism in disease vectors
Pyrethroids	Karate 5 EC (lambda-cyhalothrin)	Voltage-gated sodium channels/neurotoxic	Control of bollworms and aphids in vegetables and cotton [42]	Disease and vector control (IRS and LLINs) [43, 44]	Knock-down mutation [45] Metabolic resistance [46] Cuticle thickening [47]
Organophosphates	Dasba 40 EC (chloropyrifos)	Acetylcholinesterase (AChE) inhibitors	Insecticide against insect pests in fruits, beans, tomatoes, cotton, coffee and green vegetables [48]	Disease and vector control (IRS and LLINs) [49]	Metabolic resistance [50]
Neonicotinoids	Amekan C344 SE (200 g/l of imidacloprid and 144 g/l of cypermethrin)	Nicotinic acetylcholine receptors (n AChRs)	Systemic insecticides with contact and stomach action against sucking and chewing pests on cotton, vegetables and flowers [51].	Prequalified vector and disease control products [52, 53]	Metabolic resistance and target-sites [54, 55]
Carbamates	Farmerzeb 80 WP (80% WP of mancozeb)	Acetylcholinesterase (AChE) inhibitors	A broad spectrum protectant and preventive fungicide for the control of fungal diseases on vegetables	Disease and vector control (IRS and LLINs) [56]	Metabolic resistance [57, 58]

### Awareness and perceptions of pesticide use among agrovet store retailers

Most retailers stated that their customers were mostly rice farmers or horticulture farmers, particularly those relying on the irrigation system. The frequency of purchasing particular pesticides depended on the season. A majority of retailers reported to have no formal training on the pesticides they were selling, and poor knowledge on the type of crop pests, disease and relevant pesticides to be used for each. They were only able to recommend the use (dilution and frequency of application) based on experiences, or based on recommendations from the store owners and pesticide suppliers:

*“I have been selling pesticides for a long time. I started to work in Ifakara town shops. Also, the owner of the shop understands pesticides, and she does assist with information whenever needed”* (male retailer).

A majority of the retailers also reported giving instructions to their customers on pesticide usage, dosage and application time. However, upon examining the pesticide labels, the dosage suggested by the retailers was sometimes higher or lower than those recommended by the manufacturers on the product label. The handling of pesticides was commonly practised without protective measures. However, the retailers also occasionally provided information on use of protective measures such as wearing long-sleeve shirts and boots during preparation and spraying of pesticides:

*“Most of my customers do not know the dosage of chemicals to use. I tell them that quantity of chemicals depends on the size of the farm, amount and type weeds, and particular for insecticides it depends on the pest problem, if they ask me I always ask them how big their problem is, then I tell them to add 250 mls of Agroround (480* *g/l of glyphosate) to a 15* *L bucket”* (female retailer).

A total of 18 (17.5%) pesticides were commercially found repacked into small quantities in small unlabelled bottles. Decanted pesticide products were mainly targeting average income farmers who were able to afford small amounts.

### Crop calendar and pesticide usage practices

Most of the farmers reported cultivating more than one type of crop. Overall, 64.8% (421) of the farmers grew cereal crops, predominantly rice and maize, 25.8% (168) cultivated vegetables and fruits, such as spinach, cabbages and watermelon, 5.2% (34) cultivated legumes such as beans and 3.2% (27) grew other crops, such as cashew nuts and peanuts. Most farmers owned 1 to 20 ha of land. In the wet season, rice farmers prepared their land in November and December, planted in January and harvested in May or June. For the dry season (assisted by irrigation) they prepared farms starting in May, planted in June and harvested in October [29]. The irrigated farming practices used short-duration rice seeds, maturing in 4 months, while the non-irrigation farming method that depends on rainfall during wet season used long-duration rice seeds that mature within 5–6 months. The irrigated rice agro-ecosystem was reported to be prone to pest infestations, and hence, required regular insecticide applications. The farming methods also corresponded to the application patterns of various pesticides:

*“Normally in the rain season there are few pests and can easily be destroyed by rainwater. From my experience, the rice seed cultivated in rainy season is not vulnerable to pests, thus different from the swamp rice farming that relies on irrigation, without pesticides application you will not have good produces”* (female farmer).

### Knowledge and practices of farmers regarding pesticides and pesticide application

The majority of farmers (89.3%, n = 381) had no awareness of pesticides. Most farmers (54.4%, n = 316) sprayed doses of pesticides based on instructions received from the pesticide dealers, while (18.2%, n = 106) relied on personal experiences or direct observations based on the estimation of farm sizes and incidence of pests and weeds. Only (15.5%, n = 90) farmers reported that they read product labels, and only if written in the local language, Kiswahili. The rest of the farmers (11.5%, n = 67) relied on experts, such as agricultural officers or other knowledgeable sources of information about pesticide usage:

“*I always get instructions from the seller of the pesticides at the agrovet shop, but sometimes I read from the leaflet on the pesticide bottle only those written in Swahili”* (female farmer).

**Fig. 2:**
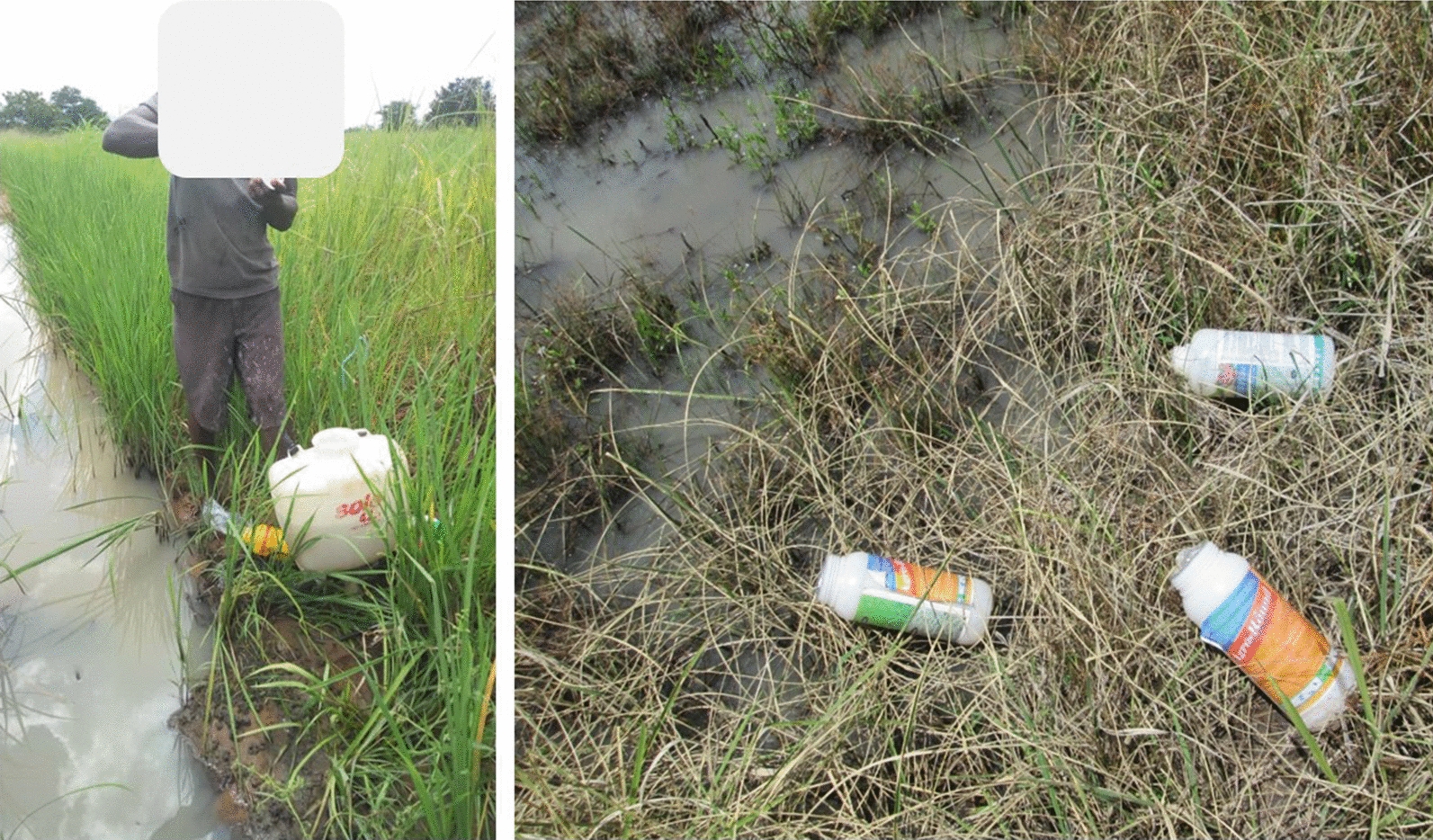
Pesticides mixing, application and disposal practices among farmers observed in rice paddies, in the study area

Only 27% of farmers believed it was necessary to use recommended pesticide doses as stipulated by the manufacturer for each pesticide, though there is no evidence that they followed those instructions. On the other hand, 62.1% perceived the right pesticide dosage as any amount enough to kill all the pests in the farm. Mixing of the pesticides was mostly done in a Knapsack^®^ Sprayer tank, traditionally recognized as “*Solo*”. Overall, 400 farmers (93.7%) performed pesticide dilutions and mixing at the farms, nearby water sources, such as irrigation canals or rivers (Fig. 2). Most of the pesticides come with the measuring equipment, but farmers typically used empty soda bottles/syringe pipe to measure liquid pesticides. Pesticide dose rates also varied among farmers (Table 5).

**Table 5:** Example of pesticide spray dosages as reported by farmers compared to the recommended dosage on the product label

Pesticide class	Trade name	Active ingredient (s)	Class of the pesticide	Knapsack spray dilution by farmers ml/l, g/l of water	Recommended knapsack dilution rate ml/l, g/l of water	Recommended dose (ml/ha)	Target crop
Insecticide	Karate 5EC	50 gm/l of lambda-cyhalothrin	Pyrethroids	15–40 ml/20 l	12 ml/20 l	300–400 ml/ha	Rice, maize, vegetables, fruits, green pepper, watermelon, beans green peas and tomatoes
	Amekan C344 SE	144 g/l of cypermethrin and 200 g/l of imidacloprid	Pyrethroids and Neonicotinoids	30 ml/20 l	8–10 ml/15 l	500 ml/ha	Tomatoes, watermelon, okra, potatoes, rice, spinach, maize, green pepper and cabbages
	Duduba 450EC	100 g/l of cypermethrin and 350 g/l of chlorpyrifos	Pyrethroids and organophosphates	30–50 ml/20 l	10 ml/20 l	400 ml/ha	Rice, cucumber, tomatoes, green pepper, cereals crops and fruits
	Buffalo 100OD	75 g/l of imidacloprid and 25 g/l of beta-cyfluthrin	Neonicotinoids and pyrethroids	35–60 ml/20 l	10 ml/20 l	500 ml/ha	Tomatoes, maize, green peas potatoes, green pepper, beans and onions
	Ninja 5EC	50 g/l of lambda-cyhalothrin	Pyrethroids	25 ml/15 l	40–60 ml/20 l	150–400 ml/ha	Rice, fruits, green peas vegetables and maize
	KungFu 5EC	50 gm/l of lambda-cyhalothrin	Pyrethroids	15–40 ml/20 l	12 ml/20 l	300–400 ml/ha	Tomatoes, watermelon, cucumber, rice, onions, vegetables, fruits and green pepper
	Suracron 720 EC/720/Profecron 720 EC	720 g/l of profenofos	Organophosphates	200–350 ml/20 l	20–40 ml/15 l	500–800 ml/ha	Cabbage and tomatoes, okra, eggplant, cucumber and watermelon
	Nogozone 60 EC	600 g/l diazinon	Organophosphates	20–40 ml/20 l	5–30 ml/15 l	150–700 ml/ha	Watermelon and cucumber
Herbicide	2,4 d Amine	720 g/l of 2, 4 d-dimethyl amine salt	Aryloxyacides	150–300 ml/16 l	200 ml/20 l	2000 ml/ha	Rice and maize
	Roundup	360 g/l of glyphosate	Amino-phosphonates	300–350 ml/15 l	200–300 ml/20 l	2000–3000 ml/ha	Rice and maize
	Parapaz 200 SL	200 g/l of paraquat dichloride	Pyridines	300–350 ml/15 l	100–200 ml/20 l	800–1600 ml/ha	Maize, rice, sugarcane and tomatoes
Fungicide	Farmerzeb 800 WP	800 g/kg of mancozeb	Dithiocarbamate	60 g/15 l	40–60 g/20 l	1000––3000 g/ha	Tomatoes, African eggplant, green pepper and potatoes
	Linkonil 500 SC	500 g/l of chlorothalonil	Organochlorine fungicide	20–50 ml/20 l	46 ml/20 l	1000–3500 ml/ha	Tomatoes, okra, eggplant, watermelon and cucumber
	Victory 72 WP	640 g/kg of mancozeb and 80 g/kg of metalaxyl	Dithiocarbamate and acylalanine	60–80 g/20 l	50 g/20 l	2000–2500 g/ha	Tomatoes, okra, and potatoes, cucumber, watermelon and cabbage

### Frequency and spraying patterns of pesticides

Most rice farmers reported re-applying insecticides at least twice every week, or anytime there were pests to achieve maximum control (Table 6). Other farmers reported preemptively re-spraying their farms to prevent pests coming from unsprayed neighbouring farms. Farmers also frequently sprayed herbicides to prevent or delay weeds:

**Table 6:** Farmers’ responses about insecticide spray frequency

Application frequency	No. of farmers	Percentage (%)
Twice every week	120	28.1
Once every 2 weeks	61	14.3
2–4 times per growing season	71	16.6
Any time I find pests in the farm	111	26.0
I do not remember	64	15.0

*“Since most of the insecticides are not as effective as they used to be, for instance, I have to re*-*apply Karate (lambda*-*cyhalothrin) two times after every week. I think it is time the effectiveness of the insecticide has depleted and cannot kill or repel pests anymore. Sometimes, I re*-*apply more often because there are a lot of insect*-*pests coming from neighbouring farms, especially those where spraying was delayed”* (male farmer).

Insecticides and fungicides were mostly used during the dry season for irrigated rice cultivation and vegetable farming. Most of the non-selective, systemic, post-emergence herbicides such as Roundup (glyphosate) were, however, sprayed before farming and planting of rice seeds, shortly before rains start during farm preparation. The selective herbicides such as 2,4-d Amine (2,4-d amine salt) were commonly used during weeding to control soft weeds in rice farms:

*“I spray Kung*-*fu (lambda*-*cyhalothrin) in the dry and wet season but mostly in the dry season because this is the period there are a lot of pests. In the wet season, there are few or no pests because of rainfall. Pest does not survive when there is a lot of water, unlike in dry season”* (female farmer).

### Challenges faced by the farmers regarding the usage of pesticides

Farmers reported multiple challenges when using pesticides. Half of the farmers (51.3%) claimed to have experienced adverse health events, such as skin irritation or coughing after spraying pesticides. The most common challenge and concern reported by about two-third of the farmers (64.6%) was that pesticides lost their killing efficiency against weeds and pests as they have had pests rebound after pesticides application. About 7.7% of the farmers suspected some pesticides are counterfeit, and 3.3% had experienced some pesticides being more diluted than expected. Switching to different classes of insecticide or mixing pesticides was a common practice (75.6% of the farmers):

*“You will find in few days sometimes even the following day after spraying there are still some pests in the farms. I surveyed and tried to spray different pesticides other than the ones I’m used to. I realized rapid attack (a mixture of cypermethrin and imidacloprid) and Amekan (a mixture of cypermethrin and imidacloprid) are far better and effective insecticides than Duduba (a mixture of cypermethrin and chlorpyrifos) alone against most of the pests affecting vegetables, watermelons and rice”* (male farmer).

### Use of pesticide mixtures

Tank mixing of more than one pesticide with the same or different active ingredients before spraying was commonly practised (Table 7), which was also observed at the farms, despite being against label instructions. Sometimes pesticides were combined with fertilisers before application following retailers’ recommendations (Table 7). The popular pesticide mixtures were: (i) two herbicides (38.7%); (ii) two insecticides (16.1%); (iii) one fertilizer and one insecticide (16.1%); (iv) one insecticide and one fungicide (12.9%); and (v) one herbicide and one insecticide (9.7%), and other mixtures (6.5%). Most farmers (86.4%) perceived cocktail sprays are more efficient than when sprayed as a single product. They also perceived that mixing two or more pesticides into a single spray solution simplified work and saved time. For example, a cocktail of KungFu (lambda-cyhalothrin) and Duduba (cypermethrin, chlorpyrifos) was used on fruits and vegetables such as watermelon, tomatoes, cabbages, okra and spinach.

**Table 7:** Pesticide combination practices by farmers at the study sites

Pesticides cocktail	Type of pesticides	Pesticide class
KungFu and Duduba	Two insecticides	Two pyrethroids and one organophosphate
2,4-D and Roundup	Two herbicides	One aryloxyacetic and one amino-phosphonates
Booster + Supercron	One fertiliser and One insecticide	Nitrogen, phosphorous, potassium and trace elements and one organophosphate
Karate and KungFu	Two insecticides	Two pyrethroids
Rapid attack and Amekan	Two insecticides	Two (pyrethroids and neonicotinoids)
Echlonil and Karate	One fungicide and one insecticide	One organochlorine fungicide and one pyrethroid
Rapid attack and Farmerzeb	One insecticide and one fungicide	One (pyrethroids and neonicotinoids) and one dithiocarbamate

### Handling and disposal practices of left-over pesticides and pesticide containers

Most farmers practised unsafe handling and disposal of pesticides. About half of the farmers (51.8%, n = 221) reported storing pesticide leftovers in their homes for either re-spraying rebounding pests or use in the next farming season. One-third (n = 128) dumped out leftover pesticides into either rivers or nearby bushes. A small minority reported burying the left-over pesticides underground (6/427) or using the pesticides to kill domestic insects such as cockroaches and houseflies in their houses (2/427). Regarding disposal of containers, the majority of farmers (55.7%, n = 238) reported that they discarded empty pesticide containers into either running water in the rivers or bushes on the farms, while approximately one-fifth (22.0%) considered burning the empty pesticides bottles. Some (18.5%) of the farmers, however, buried the containers in the ground, and a small minority (3.7%) reported washing and re-using the empty bottles for either repacking pesticides or other domestic activities.

## Discussion

Agricultural pesticides can drive selection pressure for resistance in wild mosquito vector populations breeding in agro-ecosystems [7–14], thus threatening the effectiveness of public health interventions, such as LLINs and IRS. The WHO global action plan for insecticide resistance management in malaria vectors recommends several strategies for preventing the spread of resistance, while sustaining the effectiveness of vector control interventions [26]. However, there is a lack of harmonization and integration with agricultural pesticides usage practices [8].

The current study found multiple formulations of synthetic agricultural pesticides sold at agrovet stores in the districts of Ulanga and Kilombero in south-eastern Tanzania. More than 90% of the farmers interviewed reported using either pyrethroids, organophosphates, neonicotinoids, carbamates, organochlorines or product mixtures with at least two of these classes. The active ingredients include alpha-cypermethrin, carbaryl, chlorpyrifos, chlorothalonil, cymoxanil, cypermethrin, deltamethrin, diazinon, dichlorvos, fenitrothion, imidacloprid, lambda-cyhalothrin, malathion, mancozeb, permethrin, pirimiphos-methyl, and profenofos. These insecticide groups for crop protection attack the same target sites and have similar modes of action as public health insecticides [59–61]. Most of the insecticide compounds found in use exhibit a broad spectrum of activity, indiscriminately killing even beneficial insects. These broad-spectrum insecticides are likely to be used more frequently than narrow-spectrum insecticides, thus exerting resistance selection pressure even on non-target insects, such as mosquitoes [62]. Other studies have reported extensive use of similar pesticide compounds by farmers for crop protection against pests and diseases in malaria-endemic regions [42]. For example, Philbert et al. found 48 pesticide formulations used by farmers in northern Tanzania, where malaria is endemic [63].

There are several similarities in insecticide active ingredients used in agriculture and those in public health in Tanzania. Nets impregnated with pyrethroids, mostly deltamethrin and permethrin, are widely used for malaria prevention [35]. Both lambda-cyhalothrin and bendiocarb were recently used for IRS, but have now been replaced with pirimiphos-methyl on Zanzibar Island and in some districts in north-western Tanzania [64]. Neonicotinoid-based interventions have also been tested and could be used [53]. Alpha-cypermethrin, which was found in most agricultural pesticides, is coated on Interceptor ^®^ nets, which have been under evaluation for malaria control [65]. Beyond the basic chemical similarities, public health and agricultural pesticides also share modes of actions. For example, the voltage-gated sodium channels are targeted by pyrethroids and organochlorines, while acetylcholinesterase is targeted by both organophosphates and carbamates [59, 60].

This study also revealed the presence of candidate compounds, chlorpyrifos emulsifiable concentrate (EC) and imidacloprid for both pest control on the farms and cereal preservation under storage. Chlorpyrifos, an organophosphate, was earlier recommended by the WHO Pesticide Evaluation Scheme (WHOPES) for the control of juvenile mosquitoes [66] and has been evaluated for net impregnation against mosquitoes [49]. Additionally, imidacloprid (neonicotinoids) a nicotinic acetylcholine receptor stimulator, is also being considered as an alternative or in combinations with the commonly used pyrethroids [53].

Selection pressures are experienced when mosquitoes in their aquatic stages are exposed in their breeding habitats, where most farming activities are taking place [7]. In turn, this might cause insecticide tolerance, as part of defence mechanisms that lead to insecticide resistance to a subsequent new generation of emerged adult mosquitoes [8, 10, 11, 13]. Metabolic resistance is one of the principal mechanisms in mosquitoes [67], and has been linked to the massive use of pesticides in irrigated rice plantations that enhanced the over-production of detoxifications enzymes [68]. The over-expression of metabolic genes included four CYP6P3 and one CYP325 cytochrome P450s, two delta class GSTs, one peroxiredoxin and two cuticular pre-cursor genes in adults *An.* *gambiae* s.s. collected from different breeding habitats in Benin and Nigeria was reported to be influenced by the presence of xenobiotics and agricultural pesticides in their agro-ecological sites [14, 69]. The detoxification genes and cuticular precursor genes were linked to pyrethroid resistance and reduction of insecticide penetration, respectively [69]. A study performed by Nkya et al. found that frequent exposure of *An.* *gambiae* larvae to agricultural pollutants influenced an over-expression of multiple genes responsible for the selection of target-site mutation resistance, cuticle resistance, metabolic-based resistance and nervous and synaptic-transmission based resistance in adult mosquitoes [8, 10]. Similarly, bioassays revealed that a high level of pyrethroid resistance in *An.* *gambiae* s.l. was associated with DDT and pyrethroid residues from cotton-growing farms in West Africa [16].

Glyphosate was the most common active ingredients found in most of the herbicides. However, there were also herbicides containing 2,4-dichloro phenoxy acetic acid, S-metolachlor, atrazine, paraquat and 2,4 d-amine as active ingredients. Though herbicides are generally non-toxic to insects, many of them, and also several xenobiotics, could cause metabolic stress with the potential of modifying the insecticide detoxification systems in insects, hence causing insecticide tolerance and eventual resistance [18, 20]. In one study, *Aedes aegypti* larvae exposed to glyphosate were significantly tolerant to permethrin, due to the stimulation of multiple detoxification genes, including P450s and GSTs [18].

Even though most of the agricultural pesticides found were on the list of pesticides approved in Tanzania [40, 41], there were several versions deemed of less quality but with the same brand stamp as those found in the market. These findings are in line with Shao and colleagues, who reported the magnitude of counterfeit agro-inputs in Tanzania to be as high as 46.8%, that could pose a serious risk to the ecosystem [70]. In a similar study, repacking and decanting of pesticide products in un-labelled containers was done by a quarter of pesticide dealers in six study towns in Tanzania [71]. Farmers who participated in the current study reported having experienced reduced efficacy of some pesticides, hence sprayed their crops repeatedly or at a higher quantity. Previous reports have shown the reduced effectiveness of lambda-cyhalothrin against two species of rice stem borers, mainly *Chilo* species and *Sesania calamistis* in irrigated lowland rice ecosystems in the same study area [72].

Most of the retailers of agricultural pesticides and farmers lacked formal knowledge of the proper usage of pesticides, including pesticide dosages. The majority had never been trained on agricultural pesticide usage and had a lack of knowledge of crop pest biology and disease. The retailers prescribed informal instructions to the farmers on how to apply and at what amount agricultural pesticides are required based on their experiences. The findings agree with a recent study by Lekei et al., which found that most of the retailers of pesticides in Tanzania are not qualified to provide professional instructions to the end-users [71]. Similarly, most of the farmers were not knowledgeable on crop pests and diseases, pesticide usage and management of agricultural pesticides, instead relying on information received from the retailers and personal work experience. Pesticide dilution rates were confused with application dosages and in most cases were used in larger volumes than the recommended dosage. These findings are in line with reports from southern Côte d’Ivoire, where less than half of the 208 vegetable and rice farmers who participated in a study adhered to the recommended pesticide dosage [21].

In the current study, pesticides application patterns and frequencies were observed and informed mostly by experience or perception and only to a limited extent by professional advice. Previous studies conducted in Tanzania revealed an increase in pesticide applications per season as a common practice in most farmers [73]. While the use of agricultural pesticides was influenced by the farming calendar, insecticides and fungicides were heavily used in the dry season by farmers practising irrigated rice cultivation and vegetables. Though no clear association was found on how the farming calendar influences resistance, studies in rural southern Tanzania have demonstrated clear seasonal and spatial variations in phenotypic resistance to public health pesticides in both Anopheles and *Culex* mosquito vectors, with the most resistant mosquito populations in dry seasons in areas where irrigated rice cultivations are concentrated [31, 32]. The seasonal use of agricultural pesticides might provide an opportunity for vector control programmes to partner with agriculturalists in designing a coordinated resistance management plan.

Combining two or more pesticides or with fertiliser in a spray tank was routinely practiced among farmers, mainly to enhance efficacy and to save application time (Table 7). This practice has been reported in Tanzania [63] and elsewhere [74]. Usually, different pesticide formulations are incompatible and mixing them could induce toxicity of the plant and likely influence resistance selection pressure in crop pests and even in disease vectors [21, 63].

Unsafe storage and disposal practices of left-over agricultural pesticides were reported and observed during the cross-sectional survey. Left-over pesticides were hanged on the roof or kept under the beds. Some farmers kept left-overs for the next season. However, small quantities of pesticide left-overs (i.e. generally less than a litre) were considered unwanted and were disposed either in the farms or washed off in the running water. One participant from Lupiro sprayed the left-over pesticides on the walls and the roof of the house or discarded it in the pit latrine to abate mosquitoes. The farmers also practiced unsafe disposal of empty pesticide containers. Poor storage and disposal practices of agricultural pesticides have also been reported elsewhere [75], which might pollute the ecosystem, contaminate breeding sites of mosquitoes and influence selection pressure for insecticides resistance.

This study recommends coordinated efforts between public health and agricultural sectors to prevent or delay insecticide resistance in disease vectors, while preserving the effectiveness of agricultural pesticides. The main challenge in managing insecticide resistance is not the unavailability of appropriate methods, but ensuring their adoption by farmers and pest control operators. Hence, raising awareness among pesticide retailers and farming community of the links between agricultural pesticide usage practices and insecticide resistance development in mosquitoes is urgently needed, through regular field engagement educational activities and participatory workshops and dialogues. An integrated pest and vector management (IPVM) approach could be adopted through farmer field school’s empowerment programme, in the current and future mosquito vector insecticide resistance management strategies. The adoption of principles for IPVM provides opportunities to bridge the gap between agriculture and public health. Farmers could, therefore, make rational decisions on good agricultural practices, while minimising the use of pesticides by adopting other potential pest management options that include cultural and physical control, biocontrol and the use of biopesticides.

### Study limitations

This study did not quantify the effect of agricultural pesticides in the selection of insecticide resistance in malaria vectors. Hence, there was no direct measure of association between agricultural pesticides exposure and resistance selection in malaria vectors. The study instead relied on an inventory of agricultural pesticides as well as the knowledge and practices among farmers and pesticides dealers. This research was nested in a larger study that investigated possible drivers of residual malaria transmissions [76], including insecticide resistance and resistance mechanisms in malaria vectors [27, 28], in communities where insecticidal nets are widely used, and pesticides are heavily applied in agriculture.

## Conclusions

The similarity of active ingredients in agricultural insecticides and insecticides for malaria vector control, coupled with a lack of awareness among pesticide dealers and users, might accelerate the intensity and spread of resistance in malaria vectors, thereby compromising the effectiveness of insecticide-based interventions, such as LLINs and IRS. This study emphasizes the need for improving awareness among retailers and farmers on proper usage and management of agricultural pesticides. To ensure the judicious use of pesticides and preserve the effectiveness of public health insecticides, while improving crop yields, there is a pressing need for coordinated efforts between public health and agricultural sectors in the selection, timing of application and management of pesticides. One way of achieving this goal is to initiate coordinated education programmes in elementary farmer field schools on appropriate pesticide usage in both public health and agriculture sectors. Future studies should quantify pesticide residues from the soil and water, as to better estimate the magnitude of mosquito exposures to agricultural pesticides and the impact with a view to considering integrating agricultural practices for sustainable insecticide resistance management strategies in mosquito vector populations.

## Supplementary information

**Additional file 1.** The data collection tools including the questionnaire and interview guide used in the study.

**Additional file 2.** Various pesticide classes and formulations found in the agrovet market and used by the farmers in the surveyed area.

## Data Availability

All data generated or analysed during this study are included in this published article (and its additional files).
