# Supplementary material for: Patterns of pesticide usage in agriculture in rural Tanzania call for integrating agricultural and public health practices in managing insecticide-resistance in malaria vectors
Source: Malar J. 2020 Jul 16;19:257. doi: 10.1186/s12936-020-03331-4 (PMC7364647; doi:10.1186/s12936-020-03331-4)
Supplement: Supplementary file 2 — Additional file 2. Various pesticide classes and formulations found in the agrovet market and used by the farmers in the surveyed area. [file 12936_2020_3331_MOESM2_ESM.pdf]

Survey of farmer's pesticide knowledge, usage practices and perceived effects of in mosquito vectors in Ulanga and Kilombero districts, south-eastern Tanzania

Interview Information

Date:

Name of the investigators

General data/Demographic Characteristics

Participant name/ID:

Sex

i) Male
ii) Female

What is your marital status?

a) Singe
b) Marriage
c) I don't prefer to disclose

What is your age in years?

i) 18-30
ii) 31-40
iii) 41-50
iv) 51-60

v) >60

What is your position in the community?

i) Normal citizen

ii) Hamlet leader

iii) Ward leader

iv) Agricultural officer

v) Veterinary officer

vi) Others

Where do you live?

- Name of Hamlet
- Name of Ward
- Name of District

What is your educational attainment?

i) Primary school

ii) Secondary school

iii) College/university

iv) Professional training

v) No formal training

What is/are your main economic activities?

i) Small-scale subsistence farming activities

ii) Large-scale farming for food and business

iii) Livestock keeping

iv) Small-scale business

v) Large-scale business

vi) Private employment

i) Others

Farming practices

1) Do you own a farm for farming activities?
   a) Yes
   b) No
2) Have you ever rented a farm for crop production?
   a) Yes
   b) No
   c) I don't remember
3) Where is the location of the farm?
   a) Minepa
   b) Mavimba
   c) Lupiro
   d) Mbasa
   e) Katindiuka
   f) Other farmlands
4) What is the size of the farmland used for crop production in hectares?
   a) <1 hectare
   b) 1-5 hectare
   c) 5-10 hectares
   d) >10 hectares

5) What is the type of crops you cultivated? Circle all applicable
   a) Cereals such as rice, maize
   b) Legumes such as beans
   c) Fruits and vegetables
   d) Others crops, please mention all

6) Among these, which are the rice cultivation methods you have been practising?
   a) Irrigation system i.e. Ngapa
   b) Non-irrigation on the hills
   c) Both irrigation and non-irrigation
   d) Others

Various agrochemicals and usage practices among the farmers

7) Do you know about agricultural pesticides?
   a) Yes
   b) No

8) Have you ever used pesticides in your farming activities? (if No end here)
   a) Yes
   b) No
9) How long have you been using agricultural pesticides?
   a) < 3 years
   b) 3 years' now
   c) 3-10 years
   d) More than 10 years
   e) Others, please specify years

10) Which group of pesticides among these do you use? (multiple selections)
    a) Herbicides
    b) Insecticides
    c) Fungicides
    d) Rodenticides
    e) Others (specify)
11) Which pesticides among these do you use? (refer to the checklist with all the pesticides mentioned during the in-depth interview and check out all listed/specified by the farmer)
12) Are you aware some pesticides are banned or restricted for use in farming activities?
    a) Yes
    b) No
13) Among the pesticides you have been using, are there some that have been banned or restricted for use?
    c) Yes
    d) No

14) Where do you normally get your agricultural pesticides?
   a) I purchase from the agrovet stores in my village
   b) I purchase from the agrovet stores in the city such as Ifakara town or Dar e salaam
   c) I purchase from people selling in the streets
   d) I purchase from the agricultural officer
   e) Others (please specify)

Pesticide mixing, knowledge and frequencies of application

15) Where do you conduct your pesticide mixing/dilution with water before applying?
   a) At home outdoor
   b) In the farm nearby water source
   c) In the farm far from the sources of water
   d) Others (specify)
16) How do you measure the amount of pesticides before mixing with water/ what equipment do you use to measure pesticide?
   a) I use an empty bottle of cola soda to measure pesticide
   b) I estimate the amount of pesticide by eyes
   c) I use syringe pipe to measure insecticide
   e) I use an empty pesticide bottle to measure herbicide
   f) I use the measuring equipment that is brought alongside with the pesticides
   g) I use the cover of the pesticide bottle to measure pesticide
17) Which method of application of agrochemicals do you use?
   a) Spraying after convectional mixed two or more different chemicals and water in a spray tank
   b) Spraying individual chemical mixed with water in a spray tank
   c) Sometimes I do either a or b
   d) Others, please specify
18) Do you mix and use more than one pesticide (pesticide cocktail) in a one spray tank?
   a) Yes

b) No

19) Why do you practice mixing of more than one pesticide in one spray tank?

a) Spraying pesticides in combination save time

b) Spraying pesticides in combination simplify work

c) Combined pesticides are more effective than individual pesticide

d) I don’t know

20) What are the common pesticides/products you have ever mixed in one spray tank?

a) Two herbicides

b) Two insecticides

c) One fertilizer and one insecticide

d) One fertilizer and one herbicide

e) One insecticide and one fungicide

f) One insecticide and one herbicide

g) Other mixtures (specify)

21) When do you normally spray various agricultural pesticides?

a) I spray pesticides after rainfall

b) I spray herbicides between November to January before planting rice or any other crops

c) I spray herbicides any time of the year when I see pests in the farm

d) I spray insecticide mostly in dry seasons

e) I spray insecticide only when there are pests any time of the year

f) I spray fungicide in the dry season

g) I spray fungicide in the wet season

h) I spray fungicide any time of the year

22) How often do you spray various pesticides during cropping period?

a) Once every two weeks

b) Twice every week

c) 2-4 times per growing season

d) I spray until I finish all the pesticide I purchased

e) Any time I find pests in the farm

f) Depend on the recommendation on the pesticide product label

g) I don’t remember

Knowledge, source of knowledge, storage practises of pesticides among farmers

23) Have you ever participated in any training on proper pesticide usage and management practices?
    a) Yes
    b) No
24) Do you read pesticide product label before applying?
    a) Yes
    b) No
25) Do you know the ingredients/contents of the agricultural pesticides you have been using?
    a) Yes
    b) No
26) Do you know that various pesticide has different formulations?
    a) Yes
    b) No
27) Where do you get instructions on pesticide selection, usage and storage of various agrochemicals? Please select all applicable. If the participant didn't choose **a** and **d**, please skip the next two questions.
    a) From the sellers of the agrovet stores
    b) I rely on my personal experience after using the chemicals for a long time
    c) I participated in professional training on how to use various agrochemicals
    d) From the agricultural officers
    e) I read the pesticide label information before applying pesticide, only if written in Swahili
    f) Others, please mention

28) What is the type of information or instructions gained from either the sellers of agrochemicals or agricultural officers? Please select all applicable
    a) Amount of pesticides to be sprayed
    b) Amount of water to be used for mixing pesticides
    c) Consideration of wind direction during spraying

d) The use of personal protective equipment during mixing and spraying pesticides

e) Considering the size of weeds when measuring the dosage of pesticides

f) Considering the proportion of pest infestations when measuring pesticides

g) I don't remember

h) Others, please specify

29) How much do you get satisfied with the instructions or information provided by the sellers/ and agricultural officers?

a) Highly satisfied

b) Satisfied

c) Low satisfied

d) Not satisfied at all

30) In your opinion what do you know about pesticide recommended dosage?

a) Any pesticide amount that kills all pest in the farm

b) Any quantity of pesticide that is suggested by the pesticide and requires dilution with 15-20 litres of water in a spray tank/solo

c) Half of the pesticide found in the bottle mixed with 15-20 litres of water in a solo

d) All quantity of pesticide purchased that require dilution with 15-20 litres of water

e) Amount/ rate of pesticide found on the label for a particular crop

f) Any amount of pesticide mixed with water enough to spray the farm

g) I don't know

31) Do you think it is necessary to apply the correct pesticide recommended dosage as specified on the label information?

a) Yes

b) No

c) I don't know

32) Which group of pesticides are highly used in vegetable cultivation?

a) Herbicides

b) Insecticides

c) Fungicides

d) All of the above

e) None of the above

33) Which type of pesticides are highly used in fruits cultivation?

a) Herbicides

b) Insecticides

c) Fungicides

d) All of the above

e) None of the above

34) Which type of pesticides are highly used in rice cultivation?

a) Herbicides

b) Insecticides

c) Fungicides

d) All of the above

e) None of the above

35) At which stage/period of the plant developments do you spray insecticides?

a) At nursery stage

b) Flowering period

c) Transplanting

d) Before harvesting

e) All of the above

f) Others (specify)

36) At which stage/period of the plant developments do you spray herbicides?

a) At nursery stage

b) During farms preparation

c) Flowering period

d) Transplanting

e) Before harvesting

f) All of the above

g) Others (specify)

37) How much herbicides do you use per hectare? (example if you target weeds in rice farm)

a) Less than 10mls
b) 10-40mls
c) 50-150mls
d) 150-250mls
e) 200-500mls
f) More than 500mls
g) I don't know

38) How much insecticide do you use per hectare? (example in tomatoes farm)

a) Less than 10mls
b) 10-40mls
c) 40-150mls
d) 150-250mls
e) 200-500mls
f) More than 500mls
g) I don't know

39) What do you do with the remaining/extra (both mixed/diluted or undiluted) pesticides?

a) Discard them in running water or bushes in the farms
b) Throw them in the pit latrine
c) Spray in my pit toilet to kill mosquitoes and other insects
d) Spray in my house to kill insects such as mosquitoes and cockroaches
e) Store them in the house until next farming season
f) Giveaway to my neighbor farmer to use in his/her farm
g) Bury them in the farm until next farming season
h) I had never had any remain pesticides
i) Others, please specify

40) What do you do with the empty containers after finishing the pesticides??

a) Throw them away in the running water/ bushes in the farms
b) Throw them in the bushes
c) Burn them in the farms
d) Bury them in the farms

e) Throw them in the pit latrine
f) Re-use for storage agrochemicals
g) Re-use for other household purposes
h) Others (specify)

41) Do you think there is a need to have a central station to collect leftover, old or expired agricultural pesticides?

a) Yes
b) No
c) I don’t know

What are the challenges you have been facing when using agricultural pesticides?

42) What are the challenges you have been facing when using agricultural pesticides?

a) Health effects such as skin irritation, skin burning, headache, and difficulty in breathing when mixing and after spraying pesticides
b) Pesticides are costly
c) Pesticides have a short shelf life, and they don't last long so we can keep for the next farming season
d) Some pesticides are too much diluted from the suppliers/dealers as they do not perform as expected
e) Some pesticides have lost their killing efficacy, not as previously
f) Some pesticides are counter fake
g) Pest rebound even after spraying pesticides
h) Others
i) I have never experienced any challenge since I started using pesticides

Appendix 1b:

In-depth interview guide: English version

Guide for collecting information from different farmers, who are using agricultural pesticides

Date:

Names of investigators:

Participant name/No:

Age:

Education attainment of the farmer:

Marital status:

Village/Ward/ Sub-village:

District:

Information about the farmers

1) How much land (in terms of acres) do you farm?
2) Which village or sub-village do you do your farming activities?
3) Which crop(s) do you farm?
4) Which seasons of the year do you do your farming activities? (Probe type of crops, probe on reasons)
5) Do you ever engage in the irrigation crop cultivation system? (Probe on type of crops, seasons, source of water)
6) When do you harvest your crops? (Probe on different types of crops, seasonality and farming methods)

Knowledge on different types/class of agricultural pesticides used

7) What are the agricultural pesticides do you know? (Probe on which chemicals they use, the purpose of using, effectiveness, seasonality)
8) Can you please mention all the pesticides you have been using in your farming activities?
9) Why do you use these pesticides?

10) Among the insecticides which ones do you use mostly in i) vegetable ii) rice, iii) maize iv) storage of cereals
11) At which stage/period of the crop development do you spray insecticides? Why?
12) At which stage/period of the crop development do you spray herbicides? Why?
13) At which stage/period of the crop developments do you spray fungicides?
14) Which season do you use insecticides? Why in the seasons mentioned above?
15) Which season do you use herbicides? Why in the seasons mentioned above?
16) Which season do you use fungicides? Why in the seasons mentioned above?
17) Do you know the content or ingredients of the pesticides you have been using? (probe if they read labels)

Information on purchasing of chemicals

18) Where do you normally get or purchase your agricultural supplies, including pesticides? (Probe on types and use for chemicals, preference, seasonality, amount, pesticides, herbicides, mixing)

Pesticides knowledge and usage practices

19) How do you know how to use the different agricultural pesticides you normally purchase? (Probe on the sources of information, knowledge on different types of chemicals, different types of crops, on different seasons, amount, mixing, frequency of use, considerations, herbicides, pesticides)
20) Who are your primary sources of information on how to use pesticides? (Probe on exact information given, type of chemicals, type of crops, frequency of getting information restrictions and considerations, usefulness and satisfaction of information)
21) Where do you prepare and mix pesticides before applying?
22) How do you know the correct pesticide application dosage? (probe if they read the pesticide labels)
23) How do you know pesticide formulations and, frequency of application?
24) How often do you spray insecticides and in vegetables?
25) How often do you apply herbicides in rice?
26) How often do you spray insecticide in rice?

27) Have you ever mix more than one pesticides in one spray tank before applying? If yes, why?
28) How do you know the pesticide has worked effectively?
29) After mixing and applying pesticides, how and where do you wash your pesticide equipment?
30) What challenges do you normally face when using pesticides? (probe on challenges on health, knowledge, financial, and probe on how they overcome challenges)
31) How do you usually store or dispose of the leftover diluted, and extra undiluted pesticides? (Probe on storage, disposal)
32) How do you store or dispose of empty pesticide container?

Do you have any questions/ comments regarding anything we have discussed?

Thank you very much for your time and knowledge. Just in case you have questions/concern regarding this study, please do not hesitate to contact the investigators below.

Appendix 2:

Data collecting form for sellers of agrochemical products

Date:

Name of the investigator's

Shop number (to be given by the investigator):

Name of the participant:

Location of the agrovet store, village/town:

District:

Demographic profile of the participant

1) Sex
2) How old are you (years)?
3) What is your position in this agrovet store?

4) What is your educational attainment?

5) Please specify any other income generating activities?

6) Can you explain if you have ever participating in any seminar, workshop or training on proper use and management of pesticides as a pesticide dealer? (probe if he/she has received any relevant professional training)

Type of agricultural products sold at the shop

1) What are the agricultural supplies do you sell?
2) Do you do wholesale or retail pesticide selling? (probe and observe if they decant pesticide into small containers)
3) A. What are the types of agricultural pesticides do you sell at your shop?

   B. Can I please see and photograph the pesticides that you are selling at your shop? (record the pesticides type, active ingredient in the checklist, and take a photograph for further analysis)

3. How long have you been selling pesticides?

4. Where do you purchase most of your pesticides?

Pesticide awareness and management practices

1) Who are your most frequent customers? ***(probe on different types of customers)***

2) What type of pesticides do your different customers buy? ***(probe on the different customers and what they buy)***

3) Where do most of your customers come from?

4) On average. How frequently do your customers come to purchase herbicides? (***answers in terms on times per day/week/month***)

5) On average. How frequently do your customers come to purchase insecticides? (***answers in terms on times per day/week/month***)
6)

7) On average, how much pesticides do you sell? (***answers in terms on the amount per day/week/month***)

8) Which pesticides are the most preferred/purchased and why?

9) Which pesticides group are highly demanded by rice farmers and why? (probe if there is specific pesticide type and reasons of preference)

10) Which pesticides group are highly demanded by vegetable farmers and why? (probe if there is specific pesticide type and reasons of preference)
11) Which insecticides are highly preferred by rice farmers and why?
12) Which insecticides are highly preferred by vegetable farmers and why?
13) A. Which seasons of the year do you have the highest pesticide-sales? (probe why)
    B. What pesticides are most popular in the dry season? (probe type/group of pesticides and if corresponding to the season when farmers cultivate a certain type of crop)

    C. What pesticides are most popular in the wet season? (probe type/group of pesticides and if corresponding to the season when farmers cultivate a certain type of crop)

    D. What seasons do you have the least sales?
14) What pesticides are the least popular?

15) How much on average, do people purchase different chemicals? ***(probe on the amount per person)***
16) A. Do your customers know the dosage of pesticides to use (probe if the customers ask for advice on how to use pesticides, including dosage)?

    B. If they ask you on dosage, how much do you recommend them to use? What do you consider when advising them on pesticide mixing and rates of application?

    C. Do farmers understand the pesticides information on the label? (probe if they advise them to read pesticide labels)

14) How do you dispose of the old pesticide stocks?
15) What do you do with the expired pesticides? (probe how they store/dispose them)
15) How do you store and dispose of empty pesticide containers?

Do you have any questions/ comments regarding anything we have discussed?

Thank you very much for your time and knowledge. Just in case you have questions/concern regarding this study, please do not hesitate to contact the investigators below.
